# Histopathological characteristics of cervical extensor tissue in patients with dropped head syndrome

**DOI:** 10.1186/s40001-021-00605-8

**Published:** 2021-11-26

**Authors:** Kenji Endo, Jun Matsubayashi, Yasunobu Sawaji, Kazuma Murata, Takamitsu Konishi, Toshitaka Nagao, Kengo Yamamoto

**Affiliations:** 1 Department of Orthopedic Surgery, Tokyo Medical University, 6-7-1 Nishishinjuku, Shinjuku-ku, Tokyo, 160-0023 Japan; 2 Department of Anatomic Pathology, Tokyo Medical University, 6-7-1 Nishishinjuku, Shinjuku-ku, Tokyo, 160-0023 Japan

**Keywords:** Dropped head syndrome, Histopathological characteristics, Cervical paravertebral region, Skeletal muscle, Necrosis, microvessel proliferation, Atrophy, Ligament degeneration and microvessel proliferation, Chronic phase

## Abstract

**Background:**

To date, the histopathologic characteristics of dropped head syndrome (DHS) have not been reported sufficiently. The present study investigates the histopathology of biopsy specimens from the cervical paravertebral region in patients with DHS.

**Methods:**

Histopathological parameters were evaluated in biopsy specimens of the cervical paravertebral soft tissue from 15 patients with DHS.

**Results:**

Among the 15 cases of DHS examined, skeletal muscle was identified in 7 cases, all of which showed necrosis, microvessel proliferation and atrophy. The ligament was identified in 12 cases, 8 of which showed degeneration. The lag time between the onset of symptoms and the performance of a biopsy in all 8 cases, which showed degeneration was over 3 months. Microvessel proliferation in the ligament was observed in 1 of the 4 cases, in which the lag time between the onset of symptoms and the performance of a biopsy was less than 3 months (acute or subacute phase), and in 7 of the 8 cases, in which the lag time between the symptoms and the performance of a biopsy was over 3 months (chronic phase). Chronic inflammation in the ligament was identified in 1 of the 12 cases.

**Conclusions:**

The identification of necrosis, microvessel proliferation, and atrophy in the skeletal muscle of patients with DHS and the presence of ligament degeneration and microvessel proliferation in the chronic but not acute or subacute phases may suggest that persistent skeletal muscle damage of the cervical paravertebral region causes subsequent ligament damage in patients with DHS.

## Background

Dropped head syndrome (DHS) is characterized by severe weakness of the cervical paraspinal muscles that results in the passively correctable chin-on-chest deformity [1–4]. In 1992, Suarez et al. first described four patients with DHS, which was characterized by relatively isolated neck extensor weakness, whose electromyogram and muscle biopsy results suggested a restrictive non-inflammatory myopathy predominantly affecting the cervical paraspinal muscles [5]. DHS is a relatively benign condition that may be difficult to distinguish from more ominous neuromuscular disorders presenting with severe neck extensor weakness, including myasthenia gravis, motor neuron disease, and inflammatory myopathy [1]. In 1996, Katz first described four patients with dropped head accompanied by severe neck extensor weakness in whom no specific electromyogram or muscle biopsy abnormalities were found and coined the term “isolated neck extensor myopathy” (INEM) [1, 3]. INEM is diagnosed by the exclusion of neuromuscular causes [1, 3, 4]. Although the precise etiology of DHS remains controversial, the most favored hypothesis proposes that the deformity is caused by injury to and fatigue of the paraspinal musculature with secondary kyphotic postural changes and an age-dependent loss of tissue elasticity [1, 3]. Recently, we reported the clinical study of 67 DHS patients, mainly focusing on the clinical characteristics, including the prognosis [6]. Several case reports have examined the pathological findings of DHS; these have mainly focused on skeletal muscle biopsy specimens of the cervical extensor tissue. However, to date, no large study has examined the histopathology of the cervical extensor tissue in DHS. In this study, we focused on the histopathology of the cervical paravertebral soft tissue of patients with DHS.

## Methods

The present retrospective study investigated the histopathology of the cervico-thoracic junction of 15 consecutive DHS patients who presented with correctable chin-on-chest deformity when visiting our facility between 2014 and 2020. DHS was defined as follows: (1) the patient could not maintain a neutral cervical position for even a few seconds and gradually showed chin-on chest position; and (2) the deformity was correctable in the supine position. Patients with posterior longitudinal ligament ossification and who could not maintain an upright position without assistance were excluded from the present study. All biopsy specimens were fixed in 10% neutral buffered formalin and embedded in paraffin using routine methods and then they were subjected to hematoxylin and eosin (HE) staining. Elastica van Gieson (EVG) staining and Alcian-blue staining were performed to examine fibrosis and myxoid degeneration, respectively. The specimens were then qualitatively evaluated by one pathologist (J.M.) and one orthopedic surgeon (K.E.) for the following parameters: necrosis, atrophy, microvessel proliferation, inflammation, and fibrosis in the skeletal muscle, degeneration, microvessel proliferation, and inflammation in the ligament. This study was approved by the Institutional Review Board of our university.

## Results

Table 1 summarizes the patient characteristics. Of the 15 cases examined, 1 (6.7%) was male, and 14 (93.3%) were female. The average age was 70.3 years (range 55–86 years). The clinical diagnoses of the 15 cases were as follows: isolated neck extensor myopathy (INEM) (*n* = 10), Parkinson’s disease (*n* = 1), rheumatoid arthritis (*n* = 1), uterine carcinoma (*n* = 1), lymphoma (*n* = 1), and schizophrenia (*n* = 1). Biopsy specimens were acquired by intra-operative excisional biopsy in 6 cases and needle biopsy in an outpatient clinic in 9 cases. The anatomical sites at which biopsy was performed included the cervical extensor tissues (*n* = 10) and the nuchal ligament (*n* = 5) (Table 1). The clinical summary also revealed the lag time between the onset of symptoms and the diagnosis, and the lag time between the onset of symptoms and the performance of a biopsy, in each case. Pathologically, skeletal muscle tissue was identified in 7 of the 10 cases whose cervical extensor tissues were biopsied. Furthermore, all 7 of these cases showed muscle necrosis with a ragged pattern (i.e., fiber splitting), microvessel proliferation, and atrophy. Notably, in cases 4 and 8, muscle necrosis, microvessel proliferation, and atrophy were prominent and replacement by fibrotic matrices was observed (Table 2). Chronic inflammation was observed in 2 of the 3 cases, in which the lag time between the onset of symptoms and the performance of a biopsy was less than 3 months, and also in 3 of the 4 cases, in which the lag time between the onset of symptoms and the performance of a biopsy was over 3 months (Table 3). Ligament tissue was histopathologically identified in 12 cases, 8 of which showed degeneration (Table 2). The lag time between the symptoms and the performance of a biopsy in all 8 cases that showed degeneration was over 3 months (Table 3). Microvessel proliferation in the ligament was observed in 1 of the 4 cases, in which the lag time between the symptoms and the performance of a biopsy was less than 3 months (acute or subacute phase), and in 7 of the 8 cases, in which the lag time between the symptoms and the performance of a biopsy was over 3 months (chronic phase) (Table 3). Finally, chronic inflammation was identified in 1 (case 4) of the 12 cases and ossification was identified in 1 (case 8) of the 12 cases (Table 2). The clinical presentation and pathological characteristics of the cervical extensor tissue are presented for cases 1, 3, 8.

**Table 1:** Patient background characteristics

	Sex	Age	Diagnosis	LTSD	LTSB	Method of biopsy	Biopsied tissue
1	F	80	INEM	6 M	1Y3M	Open	CET
2	F	63	RA	6 M	1Y6M	Open	NL
3	F	72	PD	6 M	5Y8M	Open	NL
4	F	59	UC	1 D	11 M	Open	CET
5	F	78	INEM	1 M	1Y5M	Needle	CET
6	M	86	INEM	3 D	10 M	Needle	NL
7	F	81	INEM	1 W	2 M	Needle	CET
8	F	55	INEM	1 D	2Y8M	Open	CET
9	F	61	Lymphoma	6 M	7 M	Needle	CET
10	F	75	INEM	1 D	2 W	Needle	CET
11	F	56	INEM	1 D	1 W	Needle	CET
12	F	72	INEM	2 D	12 M	Needle	CET
13	F	55	Schizophrenia	1 D	1 M	Needle	NL
14	F	84	INEM	7 M	1Y3M	Open	NL
15	F	77	INEM	3 D	1 M	Needle	CET

*LTSD* the lag time between the onset of symptoms and the diagnosis, *LTSB* the lag time between the onset of symptoms and the performance of a biopsy, *M* male, *F* female, *D* day, *W* week, *M* month, *Y* year, *INEM* isolated neck extensor myopathy, *RA* rheumatoid arthritis, *PD* Parkinson’s disease, *UC* uterine carcinoma, *CET* cervical extensor tissue, *NL* nuchal ligament

**Table 2:** Pathological findings of patients with DHS

	CEM (*n* = 7)					NL (*n* = 12)		
	NEC	ATR	MVP	INF	FIB	DEG	MVP	INF
1	+	+	+ +	+	−	+	+	−
2	NE	NE	NE	NE	NE	+	+ +	−
3	NE	NE	NE	NE	NE	+	+ +	−
4	+ +	+	+ +	+	+	+	+ +	+ +
5	NI	NI	NI	NI	NI	NI	NI	NI
6	NE	NE	NE	NE	NE	+	+ +	−
7	+	+	+	−	+	−	−	−
8	+ +	+ +	+ +	+	+ +	+ +	+	−
9	NI	NI	NI	NI	NI	NI	NI	NI
10	+	+	+	+	+	−	−	−
11	NI	NI	NI	NI	NI	−	+	−
12	+	+	+	−	+	+	−	−
13	NE	NE	NE	NE	NE	−	−	−
14	NE	NE	NE	NE	NE	+ +	+	−
15	+	+	+	+	+	NI	NI	NI

*CEM* cervical extensor muscle, *NL* nuchal ligament, *NEC* necrosis, *ATR*, atrophy, *MVP* microvessel proliferation, *INF* inflammation, *FIB*, fibrosis, *DEG* degeneration, *NE* not evaluated, *NI* not identified in the examined specimens

**Table 3:** Pathological findings of acute or subacute phase and chronic phase of patients with DHS

		Acute or subacute phase	Chronic phase
CEM	NEC, MVP, ATR	3 (*n* = 3)	4 (*n* = 4)
	INF	2 (*n* = 3)	3 (*n* = 4)
NL	DEG	0 (*n* = 4)	8 (*n* = 8)
	MVP	1 (*n* = 4)	7 (*n* = 8)

*CEM* cervical extensor muscle, *NL* nuchal ligament, *NEC* necrosis, *MVP* microvessel proliferation, *ATR* atrophy, *INF* inflammation, *DEG* degeneration

## Case presentation

### Case 1 (Table 1)

**Fig. 1:**
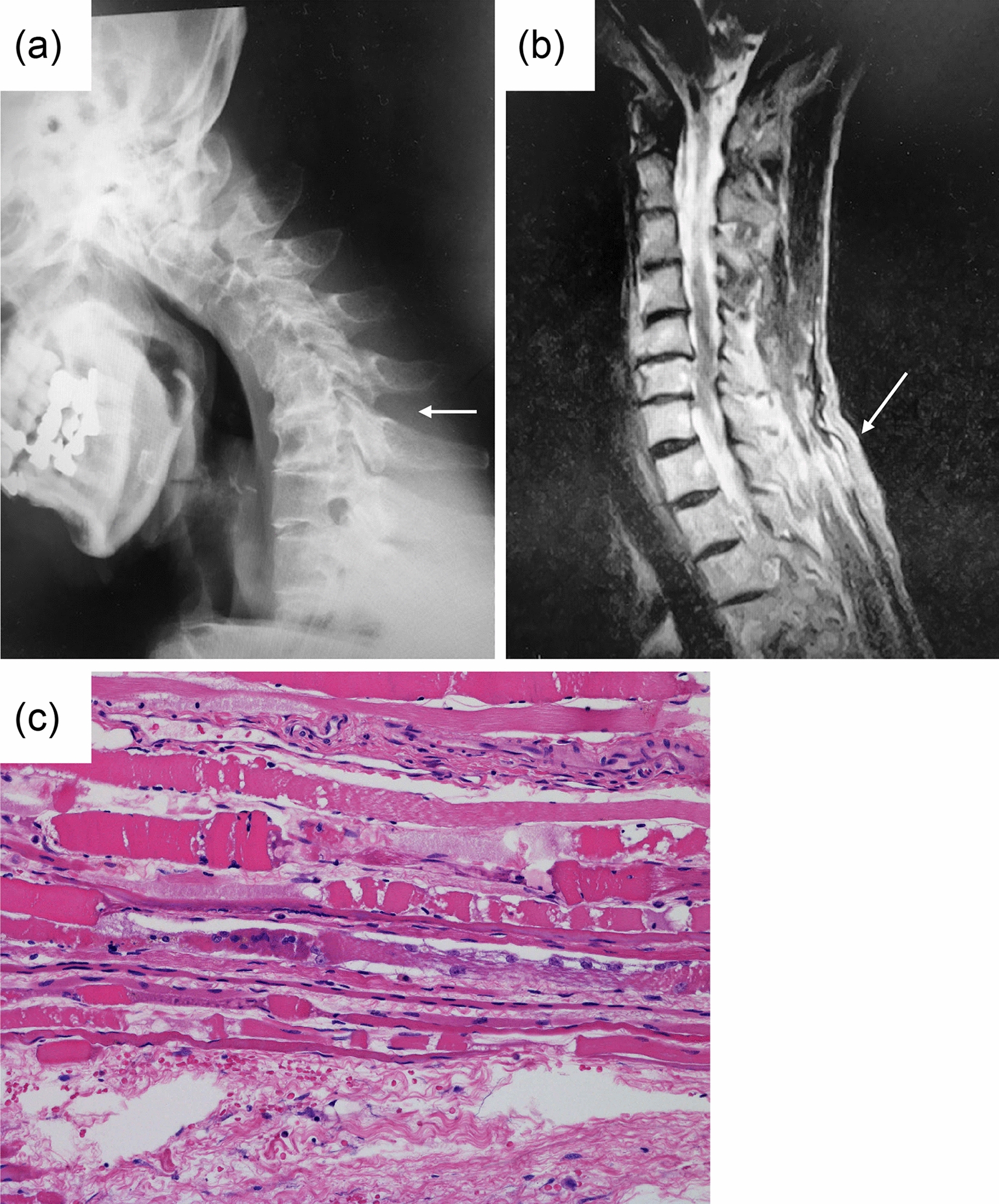
Case 1 (Table 1). **a** Lateral cervical radiographic findings (arrow indicates interspinous elongation between C6 and C7). **b** T2-weighted cervical sagittal magnetic resonance imaging (MRI) (arrow indicates interspinous high signal change). **c** Pathological findings of the interspinous tissue between C6 and C7 (hematoxylin and eosin staining)

The patient was a woman in her 80s who developed difficulty in lifting her head within a 1-month period and who also showed walking difficulty. An X-ray revealed disc narrowing and local kyphosis in C4–6 (Fig. 1a). On magnetic resonance imaging (MRI) with short time inversion recovery (STIR), a high signal intensity was present at the cervical extensor muscle of the cervico-thoracic junction (Fig. 1b). After 6 months of conservative therapy, the patient’s DHS did not improve and surgery was performed. A pathological examination revealed focal muscle necrosis with a ragged pattern, and mild microvessel proliferation (Fig. 1c).

### Case 3 (Table 1)

**Fig. 2:**
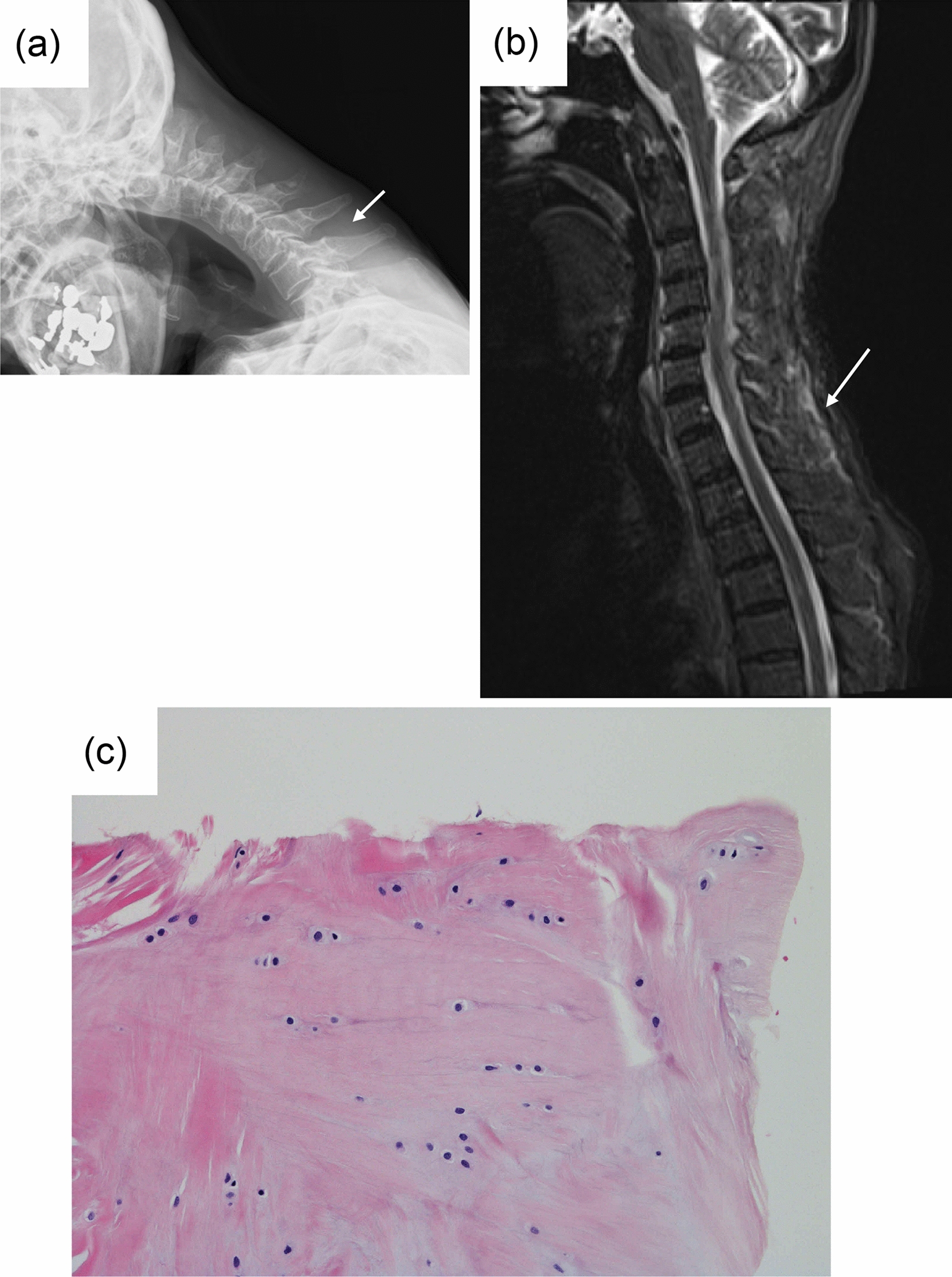
Case 3 (Table 1). **a** Lateral cervical radiographic findings (arrow indicates interspinous elongation between C6 and C7). **b** Cervical sagittal magnetic resonance imaging (MRI) with short time inversion recovery (STIR, arrow indicates interspinous high signal change). **c** Pathological findings of the interspinous tissue between C6 and C7 (hematoxylin and eosin staining)

The patient was a woman in her 70s with a history of Parkinson's disease who developed DHS. An X-ray showed interspinous space elongation in C6–T1 (Fig. 2a). On MRI, STIR showed high signal change in the cervico-thoracic junction (Fig. 2b). Surgery was performed after 6 months after the onset of symptoms. A pathological examination revealed degenerative change with chondroid metaplasia in the nuchal ligament in the cervico-thoracic junction (Fig. 2c).

### Case 8 (Table 1)

**Fig. 3:**
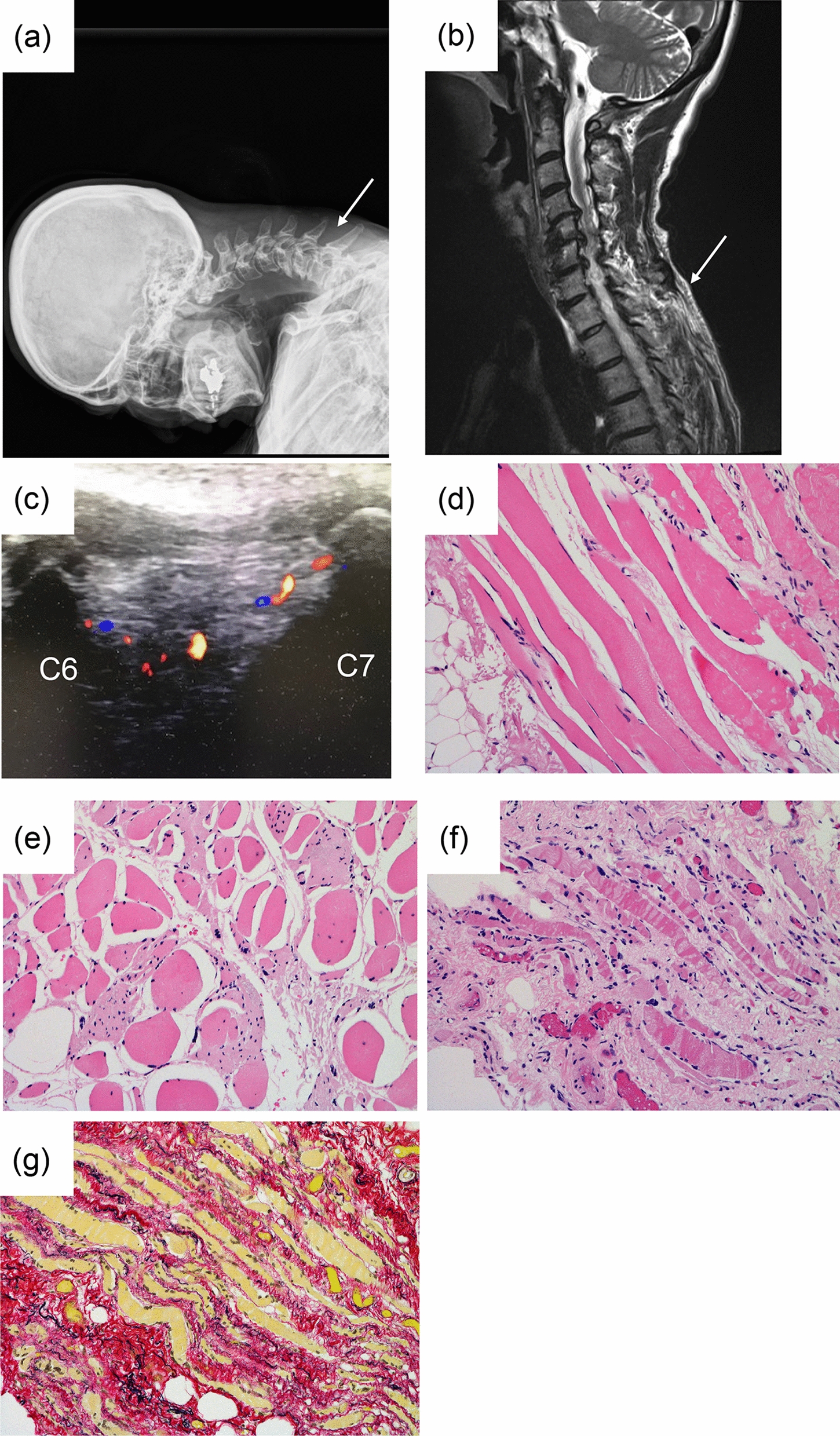
Case 8 (Table 1). **a** Lateral cervical radiographic findings (arrow indicates interspinous elongation between C6 and C7). **b** Cervical sagittal magnetic resonance imaging (MRI) with short time inversion recovery (STIR, arrow indicates interspinous high signal change). **c** Ultrasound findings suggested vascular proliferation in the interspinous muscle between C6 and C7. **d**–**g** Pathological findings of the interspinous tissue between C2 and C7 (**d** C2–3, **e** C4–5, **f**, **g** C6–7). Hematoxylin and eosin staining [**d**-**f**], Elastica van Gieson staining [**g**])

The patient was a woman in her 50s who experienced dropped head after waking up. She had been undergone cervical traction at a private clinic for 2 years. An X-ray showed interspinous space elongation in C5–T1 (Fig. 3a). On MRI, STIR showed high signal change in the cervico-thoracic junction (Fig. 3b). On ultrasound, hypervascular findings were observed at the C6–7 interspinous muscle (Fig. 3c). It gradually became difficult for the patient to walk and surgery was performed. Pathologically, although focal muscle necrosis with a ragged pattern were observed in the upper cervical paraspinal region (C2–3, C4–5) (Fig. 3d,e). There was severe muscle necrosis with a ragged pattern, with microvessel proliferation replaced by fibrotic matrices, suggesting skeletal muscle damage and atrophy in the cervico-thoracic junction (C6–7) (Fig. 3f, g).

## Discussion

To date, few reports have focused on the histopathological characteristics of DHS. Jaster et al. reported the spontaneous recovery of a patient with myopathic dropped head whose pathological findings included fiber degeneration, regeneration, and necrosis with scattered inflammatory cells, based on the examination of cervical extensor muscle biopsy specimen [7]. Suarez et al. reported that 2 of 4 patients with DHS underwent muscle biopsy of the cervical paraspinal region [5]. In one case, the pathological examination of a muscle biopsy specimen showed no evidence of inflammation. In the other case, the pathological examination of a muscle biopsy specimen showed that the muscle fibers varied in size and demonstrated the absence of inflammation. The present study revealed the histopathological characteristics of the cervical paravertebral soft tissue of patients with DHS, and notably examined the histopathology of the ligament for the first time. Necrosis, microvessel proliferation and atrophy were identified in the cervical extensor muscle without degeneration in the nuchal ligament in the acute or subacute phase (within 3 months from the onset) of DHS. On the other hand, in the chronic phase, necrosis with replacement by fibrotic matrices, microvessel proliferation, and atrophy in the cervical extensor muscle and nuchal ligament degeneration were observed (with 3 months and over). These results may suggest that persistent skeletal muscle damage of the cervical extensor region causes subsequent ligament damage in patients with DHS. In particular, the prominent fibrosis and severe muscle necrosis with a ragged pattern were considered to reveal irreversible changes in DHS, which suggests the progression of symptoms and a poor prognosis. Regarding the cervical extensor muscle in DHS cases in our study, chronic inflammation was observed in 2 of the 3 cases, in which the lag time between the onset of symptoms and the performance of a biopsy was less than 3 months, and also in 3 of the 4 cases, in which the lag time between the onset of symptoms and the performance of a biopsy was over 3 months, which might imply that persistent muscle damage due to the physical stress of DHS causes inflammation. Furthermore, pathological examinations revealed vascular proliferation in the skeletal muscle tissue of some cases of DHS; this was especially prominent in cases 1, 4, and 8. In case 8, we performed power Doppler ultrasonography, which revealed a hypervascular area in the C6–C7 interspinous muscle. As this imaging finding might be considered to be associated with the histopathological findings, power Doppler ultrasonography might be useful for the routine assessment of patients with DHS.

According to previous reports, the incidence of dropped head syndrome seems to be relatively high in elderly women [4–6, 8]. In the present study, all of the patients were ≥ 55 years of age and the M/F ratio was 1:14. As life expectancy increases, DHS will likely become more prevalent [3]. We reported that in the clinical study of 67 DHS patients, the rate of spontaneous improvement was 20.9% [6]. This result would indicate that DHS is often resistant to conservative treatment. Indeed, it has been suggested that sarcopenia can be recognized in the clinical background of DHS [9]. Sarcopenia is defined as age-associated loss of the skeletal muscle mass and function, and it is a risk factor for adverse outcomes, such as physical disability and a poor quality of life. In that study, it was reported that sarcopenia was recognized in 70% of DHS cases; in contrast, it was recognized in 25% of controls. A muscle mass decrease was noted not only in the neck muscles, but also throughout the entire body [9]. The involvement of the trunk and upper limb muscles in particular suggests a disuse atrophy of the upper body and spinal muscles. Anatomically, in the cervical extensor muscles, the multifidus and interspinal muscles are small intersegmental muscles that are inserted into the spinous processes. The semispinalis cervicis is a massive muscle originating from the transverse processes of the upper seven thoracic vertebrae [3]. Most of this muscle appeared to insert into the tips of the spinous processes of C2 and C7. The semispinalis capitis is massive muscle extending from C7 to the base, which is oriented in such a direction that the line of force generated by their contraction would result in a pure extension force on the cervical spine and head [10]. In the present study, the extensor muscles of the cervico-thoracic junction were damaged in all of the examined DHS patients; thus, weakness or laxity of the cervical extensor tissue—including the skeletal muscles—would be the main contributor to the pathogenesis of DHS [3]. Thus, from an anatomical standpoint, in addition to a clinical examination to investigate the muscle mass decrease of the entire body, a clinicopathological examination of the neck extensor tissue would be useful for evaluating the degree of progression of DHS.

## Conclusions

The identification of necrosis, microvessel proliferation, and atrophy in the skeletal muscle of patients with DHS and the presence of ligament degeneration and microvessel proliferation in the chronic but not acute or subacute phases may suggest that persistent skeletal muscle damage of the cervical paravertebral region causes subsequent ligament damage in patients with DHS.

## Data Availability

The data set used and/or analyzed during the current study are available from the corresponding author on reasonable request.
